# Urinary metabolite elimination from inhaled propylene glycol butyl ether (PGBE) and propylene glycol methyl ether (PGME) in healthy participants

**DOI:** 10.1007/s00204-026-04338-7

**Published:** 2026-03-10

**Authors:** Nancy B. Hopf, Susanne Kaspar, Jamal Bouitbir, Jennifer Pache, Sylvain Le Gludic, Gregory Plateel, Nicole Charriere, Myriam Borgatta

**Affiliations:** 1 Department for Occupational and Environmental Health, Unisanté, University Center for Primary Care and Public Health & University of Lausanne, Route de la Corniche 21, 1010 Lausanne, Switzerland; 2 Swiss Centre for Applied Human Toxicology (SCAHT), Basel, Switzerland; 3 Division of Molecular and Systems Toxicology, Department of Pharmaceutical Sciences, University of Basel, Klingelbergstrasse 50, 4056 Basel, Switzerland; 4 Forensic Toxicology and Chemistry Unit, University Centre of Legal Medicine Lausanne-Geneva, Lausanne University Hospital and University of Lausanne, Rue du Bugnon 19, CH-1011 Lausanne, Lausanne, Switzerland

**Keywords:** Neurotoxicity, Organic solvents, Toxicokinetics, Workers, Biomonitoring

## Abstract

Glycol ethers are amphiphilic organic solvents, found as mixtures of α- (secondary alcohol) and β- (primary alcohol) isomers. Propylene glycol ethers (PGE) and ethylene glycol ethers (EGE) are the most common derivatives of the glycol ethers family. Among the PGE family, propylene glycol monomethyl ether (PGME, CAS # 107-98-2) and propylene glycol monobutyl ether (PGBE, CAS # 5131-66-8) are widely used. Regulations for PGME limit the β-isomer to < 5% due to its metabolism to alkoxy propionic acid, which has been associated reproductive and neurotoxic effects similar to those observed with ethylene glycol ethers. Other PGEs do not have such restrictions despite similar isomeric composition. Propylene glycol ethers are used in many commercial products, including cleaning products, and enter the body rapidly via lungs, skin, and ingestion. We hypothesized that PGBE is metabolized to 2-butoxypropionic acid (2-BPA), as suggested by in vitro hepatocyte experiments, and that 2-BPA would be quantifiable in urine from healthy participants (*N* = 17) following a 2-h exposure to a vapor mixture containing 15 ppm PGBE and 35 ppm PGME. Metabolites were quantified with liquid chromatography with quadrupole mass spectrometry (LC-MS/MS). Our in vitro hepatocyte experiments showed that PGBE was metabolized to 2-BPA. Urinary metabolite concentrations from the participants were greater for PGBE compared to PGME, and their peak elimination occurred at 1 h post exposure. Our findings show that β-isomers in commercial propylene glycol ethers can readily metabolize to the corresponding alkoxy propionic acid. However, current toxicological hazard assessments are insufficient to evaluate possible health implications.

**Supplementary Information:**

The online version contains supplementary material available at https://doi.org/10.1007/s00204-026-04338-7.

## Introduction

Glycol ethers are common ingredients in a wide range of products, including paints, adhesives, coatings, degreasing agents, dyes, polymers, and other common products (van der Hoek et al. 2000). Although some glycol ethers have been associated with neurotoxicity in animal studies (ECETOC 2005) and in workers exposed to high levels in occupational settings (Morton 1990), organic solvents such as glycol ethers are not systematically tested for neurotoxic effects. The European Union (EU) legislation does not require neurotoxicity testing, and to date, no specific animal studies have been conducted to assess the potential neurotoxic effects of organic solvents in general (ECETOC 2005).

In this context, the propylene glycol ether family has not been extensively tested for neurotoxicity; however, studies on ethylene glycol ethers have shown the activation of some several neurotoxic mechanisms, including altered antioxidant capacity, enhanced lipid peroxidation, and increased caspase-3 activity in the frontal cortex and hippocampus of rats (ECETOC 2005). Their neurotoxicity increases with lipophilicity, and adverse effects on central nervous system cells may contribute to the pathogenesis of neurodegenerative disorders (Pomierny et al. 2013). The neurotoxicity observed in animals exposed to ethylene glycol ethers were not attributed to the parent compound, but rather to the alkoxy acid metabolites (ECETOC 2005).

**Fig. 1:**
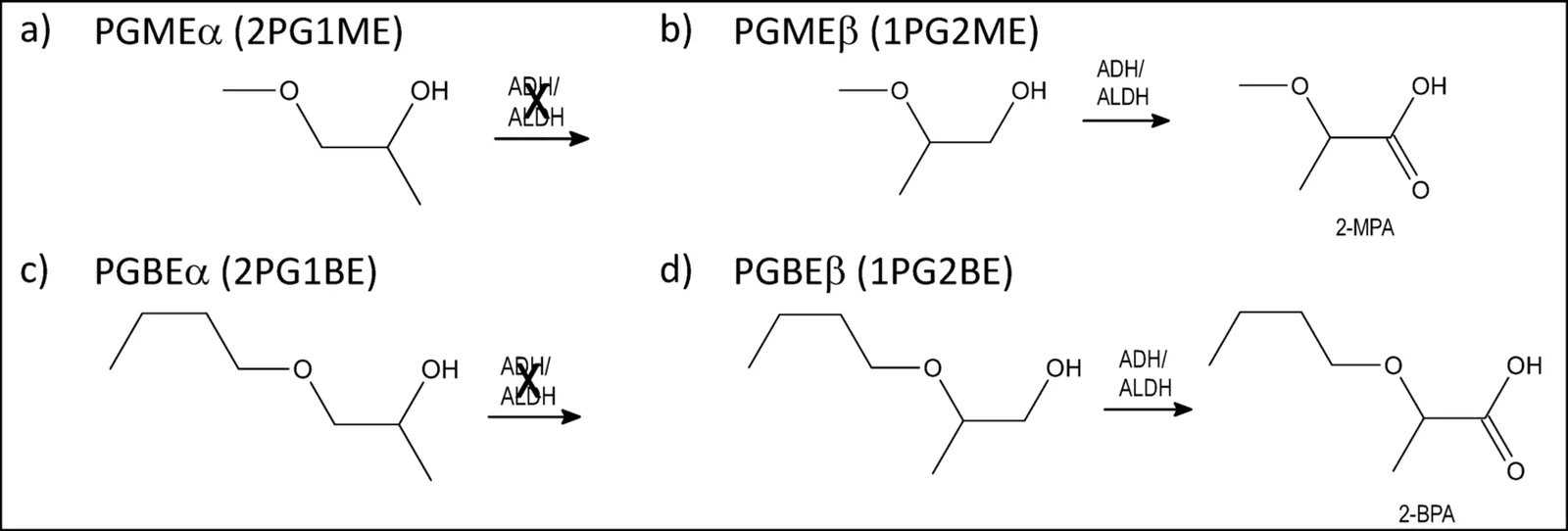
Structural isomers of propylene glycol ethers. **a** Propylene glycol monomethyl ether (PGME) isomers with the secondary alcohol group (major isomer). **b** PGME with the primary alcohol group (minor isomer—less than 5% in technical grade solvent) that can be oxidized by alcohol dehydrogenase (ALD) followed by aldehyde dehydrogenase (ALDH) to the corresponding alcoxyacid 2-methylpropyl carboxylic acid (2-MPA9. **c** Propylene glycol monobutyl ether (PGBE) with a secondary alcohol group (major isomer). **d** PGBE with a primary alcohol group (minor isomer – fraction in technical grade unknown) that can be oxidized by ALD followed by ALDH to 2-butoxypropionic acid (2-BPA)

Commercial propylene glycol ethers are typically mixtures of isomers: the α- (secondary alcohol) and β- (primary alcohol) isomers. The primary alcohol group in the β-isomer can be oxidized by alcohol dehydrogenase to the corresponding aldehyde, and further to the corresponding alkoxy propionic acid by aldehyde dehydrogenase (Fig. 1). These enzymes are present not only in the liver, but also in the lungs and skin. Notably, propylene glycol monomethyl ether (PGME) regulation stipulates that the commercial mixtures should contain less than 5% of the β-isomer to reduce possible health risks. However, no such restrictions exist for other propylene glycol ethers even though they are also produced as isomer mixtures.

The concentrations of α- and β-isomers in the brain following exposure remain unknown, raising concerns about their potential neurotoxic effects. To address this gap, toxicokinetic (TK) models have been used to predict brain concentrations of glycol ethers (Reale, 2023), focusing particularly on two widely used compounds: PGME (CAS # 107-98-2) and propylene glycol monobutyl ether (PGBE; CAS # 5131-66-8).

The TK model showed that brain concentrations of both compounds increase proportionally with exposure levels (Reale, 2023). While PGME currently has an occupational exposure limit, no such limit exists for PGBE. Both PGME and PGBE are quickly absorbed through the lungs into the bloodstream following inhalation (Borgatta et al. 2022). Additionally, dermal absorption represents a significant route of exposure, as glycol ethers readily cross the skin barrier (Berthet et al. 2020). Once absorbed, propylene glycol ethers are distributed throughout the body via the bloodstream, either bound to plasma proteins or in free form, and can passively diffuse across biological membranes, including the blood-brain barrier (BBB) (Reale, 2023). Exposure to glycol ethers may occur both at home and in occupational settings, as demonstrated by biomonitoring studies involving adults and children (Béranger et al. 2017; Binter et al. 2019; Garlantézec et al. 2020). Therefore, it is essential to monitor exposure in individuals handling products containing these organic solvents, especially given the absence of regulatory limits and insufficient neurotoxicity data. To this end, human biomonitoring (e.g., in urine) is the preferred method to quantify internal exposures to propylene glycol ethers, which can enter the body via several routes including inhalation and skin absorption from several sources. An additional advantage for using biomonitoring is that propylene glycol ethers have high blood: air partition coefficients (Johanson and Dynésius 1988), and consequently, an increased physical activity such as cleaning and painting will result in dramatically increased uptake rates (De Luca et al. 2025; Laitinen et al. 2006). Urine collection is a convenient sampling method for quantifying exposure biomarkers in the body (internal exposure). While 24-h urine collection provides the most comprehensive assessment of total internal exposure, spot urine samples are much more practical for large population studies. However, the timing of the spot urine sample is critical; it should coincide with the expected peak urinary concentrations to accurately estimate the total body burden. TK studies of glycol ethers determine the time interval between the exposure peak and maximum urinary concentrations (Devanthéry et al. 2000, 2002; Hopf et al. 2012; Tomicic et al. 2011).

Controlled human experiments using exposure chambers with known air concentrations of glycol ethers are employed to elucidate their toxicokinetics, including peak urinary elimination concentrations. Urinary PGME serves as a specific exposure biomarker and follows an exponential decay with a short half-life of approximately 2–3 h (Devanthéry et al. 2002; Hopf et al. 2012; Tomicic et al. 2011). The ß-isomer of PGME (PGMEß) is metabolized to the corresponding 2-methyl propionic acid (2-MPA) (ECETOC 2005). Although no formal TK study has yet been performed for the ß-isomer of PGBE (PGBEß), its supposed metabolite, 2-butyl propionic acid, has been quantified in the general populations (Fromme et al. 2013).

Based on these considerations, we hypothesized that the PGBEß isomer in technical grade PGBE would be metabolized in vitro by human hepatoma cell line HepaRG cells to the corresponding metabolite, 2-butoxypropionic acid (2-BPA), and that this biotransformation would be sufficiently extensive to allow quantification of 2-BPA following inhalation exposure to low concentrations of technical grade PGBE in healthy participants exposed under controlled conditions. Similarly, we hypothesized that PGMEß would be metabolized in vitro by HepaRG cells to 2-MPA, and that this metabolite could be quantified in urine after inhalation exposure to PGME in the same participants. Furthermore, we hypothesized that the previously developed TK model for PGBE could accurately predict urinary 2-BPA concentrations by incorporating this metabolic pathway. Our study goals were to: (1) quantitatively compare the formation of 2-BPA and 2-MPA metabolites in differentiated HepaRG cells in vitro following exposure to technical grade PGME (containing less than 5% of the ß-isomer) and PGBE (with an unknown ß-isomer content, as there are no regulatory limits on this), and (2) determine the urinary elimination profiles of 2-MPA and 2-BPA in a controlled human exposure experiment where healthy participants were exposed to known air concentrations of technical grade PGME and PGBE, either individually or as a mixture.

## Methods

### Chemicals and materials

PGME and PGBE commercial products (i.e., technical grade solvent) were used in this study. PGME (CAS # 107-98-2, ≥ 99%, CAS # 1589-47-5, < 0.5%), PGBE (CAS # 5131-66-8, ≥ 99%), the internal standard (IS) propylene glycol propyl ether (PGPE, CAS # 1569-01-3, 99%) as well as the metabolites 2-MPA (CAS # 1589-47-5), 2-BPA (CAS # 5131-66-8), and 2-propoxypropionic acid (2-PPA, IS) were obtained from Sigma Aldrich (Buchs SG, Switzerland), sodium sulphate Na_2_SO_4_ (CAS 7757-82-6) from Merck (Darmstadt, Germany). Solid Phase Extraction (SPE) cartridges were bought from Chromabond HR-XA, 85 μm, 200 mg/3 mL, Macherey-Nagel, Oensingen, Switzerland), syringe filters (4 mm PTFE syringe filters (hydrophilic, 0.45 μm, pk.200) from BGB Analytik, Boeckten, Switzerland), and an evaporation manifold system (TurboVAP, Caliper TurboVap LV Evaporator, Marshal Scientific, New Hampshire, USA) were used for sample preparation. Isoniazid, β-naphthoflavone, and phenobarbital were bought from Merck (Darmstadt, Germany).

### In vitro metabolism using HepaRG cells

We used the well-characterized human hepatoma cell line HepaRG cells purchased from Thermo Scientific (Wohlen, Switzerland), which mimic mature hepatocytes (Guillouzo et al. 2007). The main advantage of HepaRG cells is the reproducibility of their results; this is not the case with different batches of cryopreserved human hepatocytes (Duivenvoorde et al. 2021; Gerets et al. 2012). Cells were cultured and differentiated as described before (Grünig et al. 2017; Guillouzo et al. 2007). Briefly, HepaRG cells were initially cultured in William’s E medium (Thermo Fisher Scientific, Waltham, MA, USA) supplemented with 10% fetal bovine serum (FBS) (Biopredict International, Saint-Gregoire, France), 100 U/mL penicillin, 100 µg/mL streptomycin, 2 mM glutamine, 5 µg/mL insulin (Bioconcept, Allschwil, Switzerland), and 50 µM hydrocortisone hemisuccinate (Sigma-Aldrich, Buchs, Switzerland). After two weeks, differentiation was induced by continuing culture in the same medium supplemented with 1.7% DMSO for an additional two weeks to promote expression of liver-specific functions (Allard et al. 2021). Throughout this one-month cultivation period, the medium was refreshed three times per week. Cells were maintained at 37 °C with 5% CO_2_ in a humidified atmosphere. For cytochrome P450 (CYP) induction fully differentiated HepaRG cells were kept additional three days in low FBS (2%) medium, containing the CYP-inducers isoniazid (50 µM), β-naphthoflavone (25 µM), or phenobarbital (500 µM), with daily medium change, to explore further metabolism of 2-BPA in vitro. HepaRG cells were treated with increasing concentrations of technical grade PGME (Sigma Aldrich) (from 0 up to 1000 mg/L) or with increasing concentrations of PGBE (0 up to 200 mg/L) in the presence of CYP inducers for 24 h. After the treatment, supernatants for each condition were collected and were used to quantitatively compare the formation of propylene glycol ether metabolites by alcohol dehydrogenase (ADH) and aldehyde dehydrogenase (ALDH).

### Healthy participants

Participants (*N* = 17) in our study were the same volunteers recruited and screened in Borgatta and colleagues (Borgatta et al. 2022). Of the 17 participants, six were women and 11 men. The mean age of participants was 31 years, with a standard deviation of 13 years. Women had a mean age of 34 years, while men averaged 29 years. The mean body mass index (BMI) was approximately 24 kg/m² for both sexes, with a standard deviation of 3.

Recruitment was conducted via flyers distributed at the University of Lausanne and the Lausanne University Hospital (CHUV), as well as through email invitations sent to individuals who had participated in previous research projects. Interested individuals were invited to the University Center for Primary Care and Public Health (Unisante), University of Lausanne, where they underwent a comprehensive screening session to verify health status. This included anamnesis, blood analysis (e.g., liver and kidney function), electrocardiogram, spirometry, and BMI assessment. Eligibility criteria required participants to be over 20 years of age, non-smokers, not occupationally exposed to PGME or PGBE, and, among women, not under hormonal regimen. A pregnancy test was administered to non-menopausal women prior to each exposure session. All screening results were within clinical limits, and pregnancy tests were negative. Participants were instructed to abstain from alcohol consumption the day before each exposure to avoid metabolic interference. They were reimbursed for their time and inconvenience. All participant signed a written informed consent according to the human ethics committee of canton Vaud (Commission cantonale d’éthique de la recherche sur l’être humain), which approved the study (amendments to the PB_2017-00043 (343/14) project).

### Controlled exposure with healthy participants

Health adult volunteers participated in a controlled exposure study conducted in a 12 m^3^ exposure chamber specifically designed for inhalation experiments. The experimental setup has previously been described (Borgatta et al. 2022). Briefly, each participant underwent three separate 2-h exposure sessions: one with PGME alone, one with PGBE alone, and one with a mixture of both solvents. The sequence of exposure was randomized, and a minimum interval of one week was maintained between sessions to minimize potential carryover effects. Air concentrations of PGME and PGBE were set below the Swiss occupational exposure level (OEL) for PGME (i.e., 50 ppm) with PGME concentration at 35 ppm (131 mg/m^3^) and PGBE 15 ppm (83 mg/m^3^). These levels were chosen to reflect realistic occupational scenarios while ensuring participant safety. Airborne concentrations of PGME and PGBE were continuously monitored with a photoionization detector (PID) interfaced with a software (Lab VIEW software, National Instruments Corporation, Texas, USA), providing real-time feedback and control. In addition, air samples (Anasorb 747 sorbent tubes according to National Institute for Occupational Safety and Health Manual of Analytical Methods # 2554) were collected during exposures for all sessions and analyzed to confirm the targeted exposure concentrations. Participants remained seated and at rest during the exposure sessions to minimize variability in respiratory uptake. The exposure chamber was thoroughly ventilated between sessions to prevent cross-contamination.

### Chemical analytical analysis

The samples (urine from participants and cell culture media from the HepaRG experiments) were loaded onto the conditioned SPE cartridge after enzymatic deconjugation of the metabolites (glucuronidase enzyme (incubated overnight) and 2-PPA as the internal standard), eluted with an acidified methanol solution (1% acetic acid), filtered, evaporated until dry (52 °C, 3 psi for 30 min), and reconstituted in ammonium acetate buffer (15 mM, 1 mL). The sample (50 µL) was injected into an ultra-performance liquid chromatography (UPLC) (Dionex Ultimate 3000 system, Thermo Scientific, Reinach, Switzerland) with a silica column (maintained at 40 °C, Acquity UPLC HSS T3, 1.8 μm, 2.1 × 150 mm, Waters, Baden Dätwill, Switzerland) coupled with a tandem mass spectrometry (TSQ Quantiva, Thermo Scientific) equipped with an electrospray ionization source (ESI) and operated in negative ion mode (2350 V). The mobile phase (A: ammonium acetate buffer (15 mM) and B: acetonitrile) program was: t = 0 min: 0% B, t = 2.5 min: 70% B, t = 3 min: 70% B, 3.1 min: 0% B, at a flow rate of 300 µL/min (total cycle time of 9 min) giving retention times for 2-MPA at 1.80 min, 4.84 min for 2-PPA and 5.24 min for 2-BPA. Calibration curves were prepared by diluting 2-BPA and 2-MPA in synthetic urine with minimum six calibration points ranging from 2 to 5000 ng/mL. Limit of detection (LOD) was 2 ng/mL and limit of quantification (LOQ) was 5 ng/mL.

### Statistical analysis

Comparative and descriptive analyses were conducted using RStudio software (www.rstudio.com, RStudio 2013, version 2023 − 06.1 + 524) with the statistical language R (http://cran.r-project.org) (R Core Team, 2023). Differences were considered significant for *p* ≤ 0.05.

## Results

### In vitro HepaRG metabolism

**Fig. 2:**
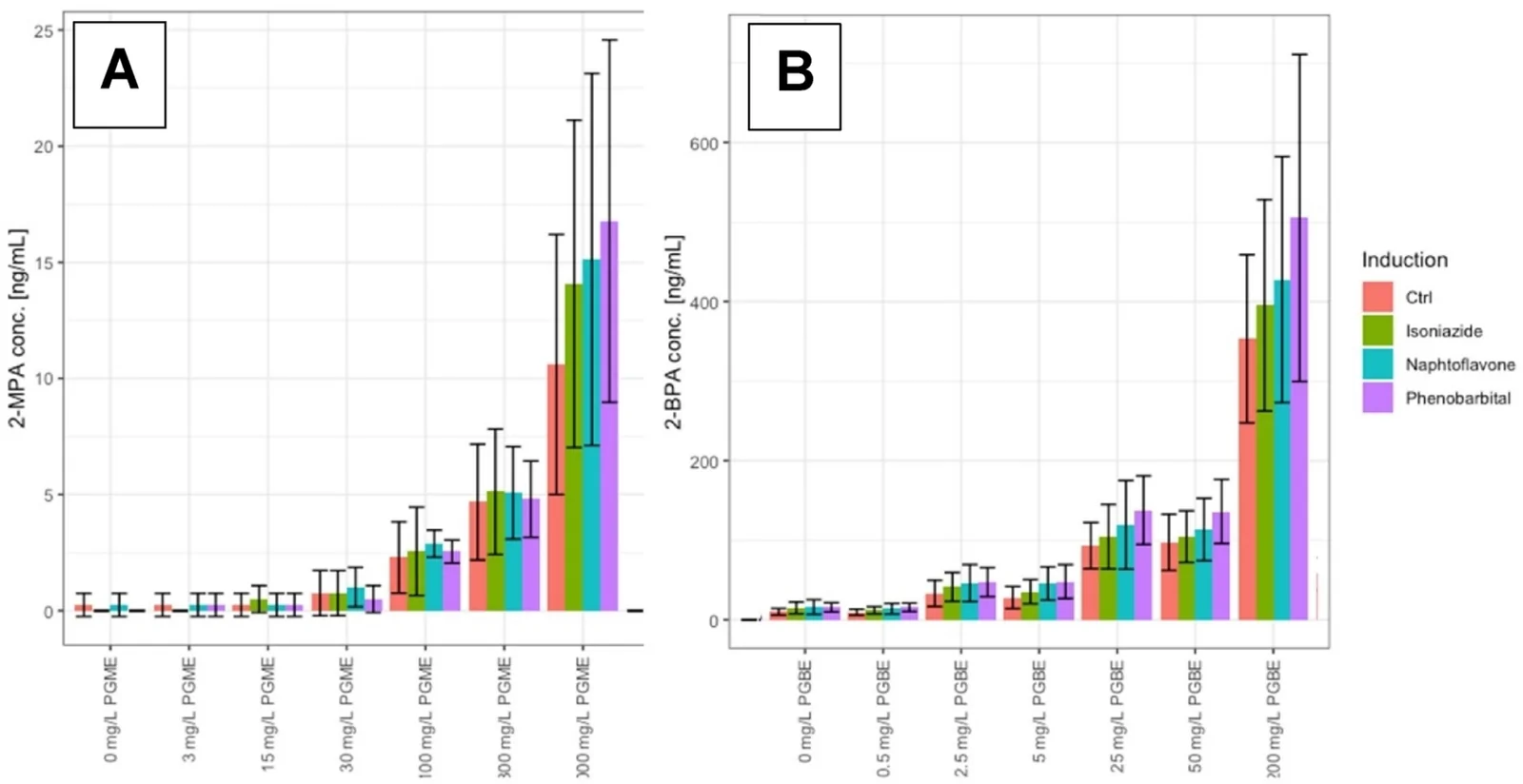
Metabolite concentrations produced in differentiated HepaRG cells exposed to propylene glycol ethers for 24 h. Differentiated HepaRG cells were treated for three days with 5 µM isoniazid, 25 µM β-naphthoflavone, and 500 µM phenobarbital to explore the involvement of specific CYP enzymes. **A** 2-MPA concentrations measured in supernatant after exposure of HepaRG cells to PGME up to 1000 mg/L. **B** 2-BPA concentrations measured in supernatant after exposure of HepaRG cells to PGBE up to 200 mg/L. The y-axes have different scales for each metabolite. Results are presented as mean ± standard deviation (SD) of four independent experiments (*n* = 4)

Cell viability, evaluated by intracellular ATP content and membrane toxicity assay, remained unchanged in HepaRG cells after 24 h exposure to PGME (up to 1000 mg/L) and PGBE (up to 200 mg/L), regardless of CYP modulator treatment (Figure S1A–D). Then, we assessed the concentrations of metabolites, namely 2-MPA and 2-BPA, produced by HepaRG cells after exposure to PGME and PGBE for 24 h, both with and without metabolic induction. Only minimal concentrations of 2-MPA were detected across all experimental conditions (Fig. 2A), whereas 2-BPA levels reached up to 540 ng/mL with a standard deviation of 100 in the highest PGBE treatment group (200 mg/L) (Fig. 2B). Figure 2B shows that 2-BPA metabolite formation in HepaRG cells was primarily influenced by the concentration of PGBE, with higher exposure levels corresponding to increased metabolite production across all induction conditions. These results support that HepaRG cells in vitro effectively replicate the specific metabolic pathways of propylene glycol ethers (PGEs).

Next, we examined the formation of 2-BPA in HepaRG cells under treatment with cytochrome P450 (CYP) enzyme modulators using isoniazid, β-naphthoflavone, and phenobarbital to explore the involvement of specific CYP enzymes in this biotransformation. Isoniazid, a competitive inhibitor of CYP2C19, CYP3A4, and CYP2A6, a stabilizer of CYP2E1 (Hakkola et al. 2020; Wen et al. 2002), had no significant effect of 2-PBA levels at either tested PGBE concentration. Although induction verification showed a slight but significant increase in *CYP2E1* mRNA expression (Figure S2B), this result suggests that CYP2E1, CYP2C19, CYP3A4, and CYP2A6 are not involved in the formation or further metabolism of 2-BPA in this system.

β-Naphthoflavone is a CYP1A1 and CYP1A2 inducer in primary hepatocytes and mice (Chang et al. 2017; Chatuphonprasert et al. 2011; Park et al. 2019; Zhang et al. 2003). Upon treatment with β-naphthoflavone (25 µM), 2-BPA levels were significantly reduced to 227 ± 100 ng/mL in comparison to 541 ± 50 ng/mL in control conditions (Table S2). The increase in *CYP1A1* mRNA expression was confirmed in HepaRG cells following exposure to β-naphthoflavone, showing a significant approximately 300-fold induction (Figure S2A). The marked reduction of 2-BPA levels upon β-naphthoflavone treatment suggests that CYP1A1 and/or CYP1A2 may be involved in the further metabolism or degradation of 2-BPA, possibly through O-dealkylation, a known function of these enzymes (ECETOC 2005).

**Fig. 3:**
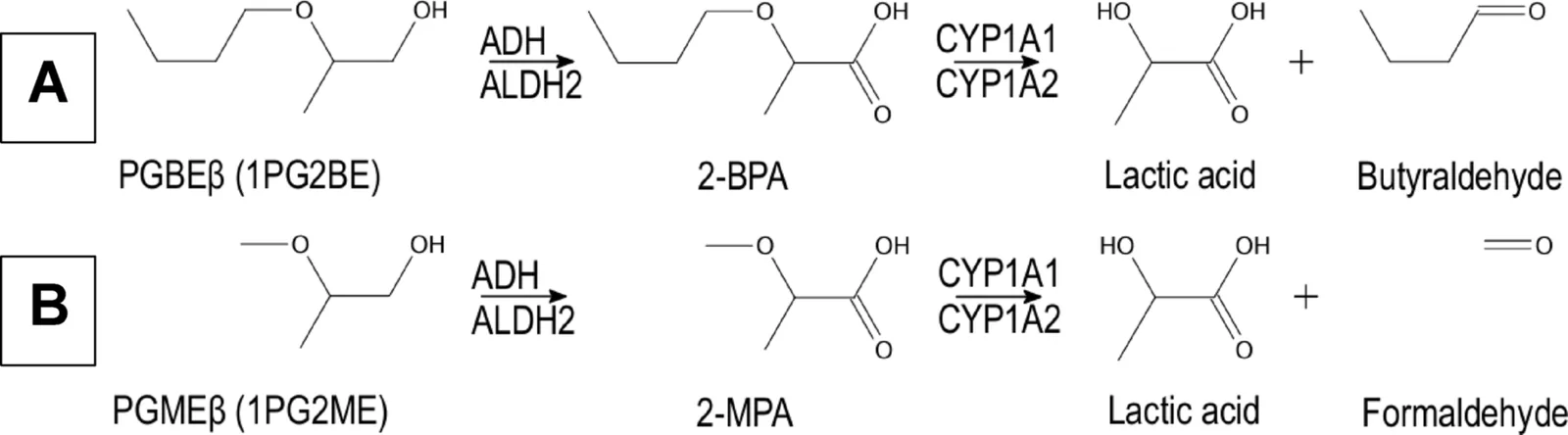
Proposed metabolic pathways for PGBEβ and PGMEβ. **A** Degradation of PGBEβ to 2-BPA, followed by CYP1A1/CYP1A2-mediated conversion to lactic acid and butyraldehyde. **B** Degradation of PGMEβ to 2-MPA, followed by CYP1A1/CYP1A2-mediated conversion to lactic acid and formaldehyde

Phenobarbital, an indirect CAR activator and broad CYP inducer (notably CYP3A4, CYP2B6, and others) (Hakkola et al. 2020), also did not alter 2-BPA levels compared to control. HepaRG cells showed an approximately 300-fold significant increase of mRNA expression of *CYP1A1* (Figure S2A) and an approximately 15-fold significant increase of *CYP2B6* mRNA expression ((Figure S2C). Despite this induction, the lack of metabolic impact suggests that CYP3A4 and CYP2B6 are likely not significantly involved in the formation or clearance of 2-BPA under the tested conditions. Figure 3 shows the proposed metabolic pathways for PGBEβ and PGMEβ to their to their respective metabolites via CYP1A1/CYP1A2-mediated conversion to lactic acid and aldehyde.

### Urinary elimination of metabolites

#### Urinary metabolite concentrations before and after two hours of exposure

**Fig. 4:**
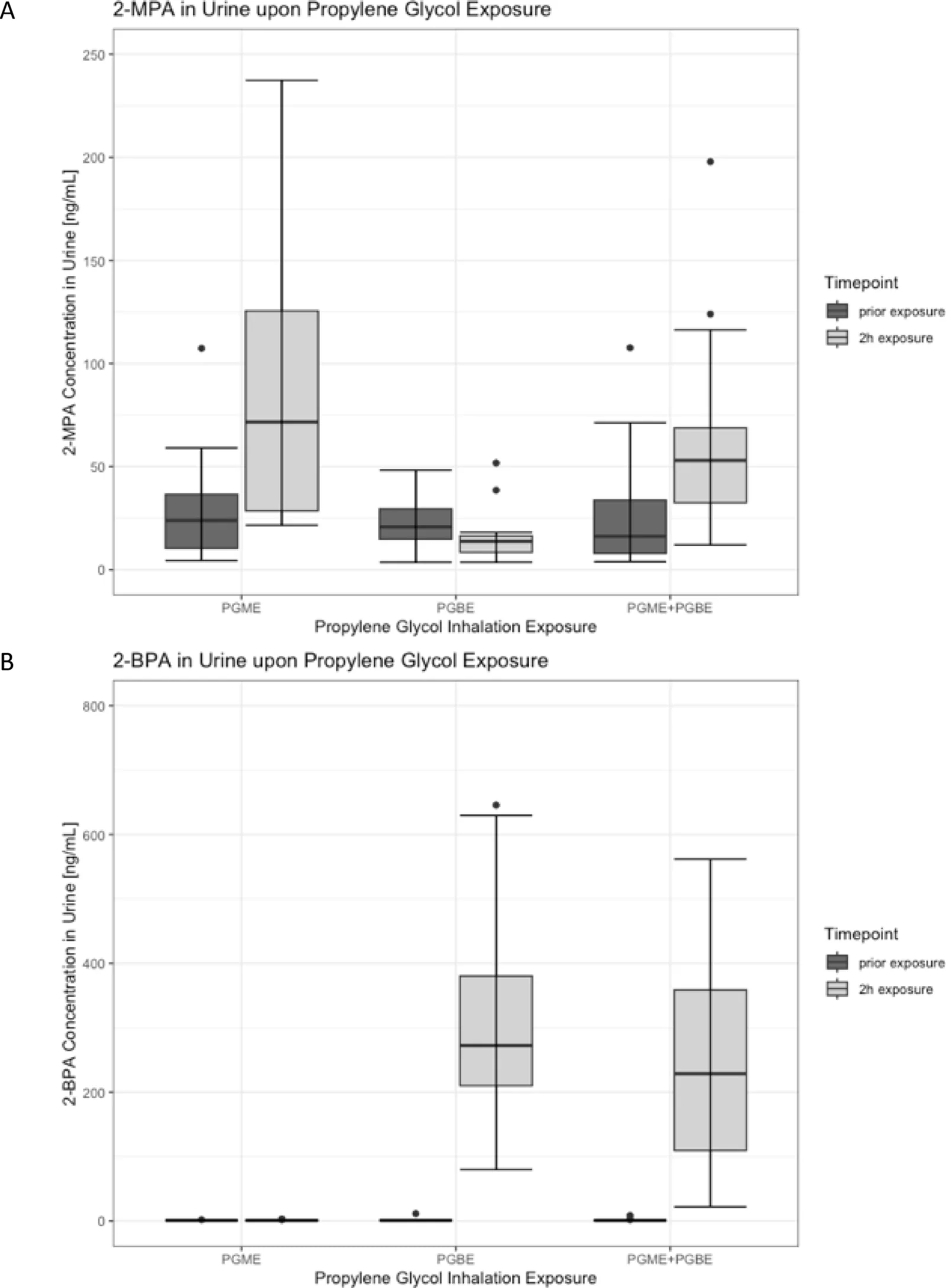
Urinary concentrations of metabolites following PGME, PGBE or PGME-PGBE mixture inhalation exposure. **A** 2-MPA [ng/L] and **B** 2-BPA [ng/L] after exposure to PGME, PGBE, or a PGME–PGBE mixture. Data are presented as boxplots, showing median, interquartile range, and outliers

To evaluate the effects of controlled exposure on urinary metabolite levels, we analyzed pre- and post-exposure samples from 17 healthy participants. Figure 4 shows the boxplots of the mean and standard deviations for each urinary metabolite before and after the exposure sessions. The urinary metabolite 2-MPA was quantified immediately after 2 h of exposure to PGME as well as after the PGME-PGBE mixture. One participant showed particularly high baseline levels of 2-MPA (maximum of 702 ng/mL; > 20 standard deviations above average) and unusual response patterns across all investigated urine metabolites. These data were treated as outliers and excluded from further investigation.

Inhalation exposure to the same PGME concentration either alone or as a PGME-PGBE mixture led to a marked increase in urinary 2-MPA concentrations compared to baseline with mean concentrations of 95.2 ± 20 ng/mL and 63.3 ± 15 ng/mL, respectively (Fig. 4A). Likewise, inhalation exposure to PGBE alone or PGME-PGBE mixture led to significant increase in urinary 2-BPA concentrations with mean concentrations 304.6 ± 120 ng/mL and 251.4 ± 100 ng/mL, respectively (Fig. 4B).

Urinary 2-MPA concentrations were consistently detected in all urine samples (mean concentration: 15.75 ± 5 ng/mL) before the exposure sessions. Background concentrations of 2-BPA (1.27 ± 0.5 ng/mL) were very low and far below 2-MPA concentrations.

#### Preliminary experiment for urinary elimination curves for 2-BPA in two researchers

**Fig. 5:**
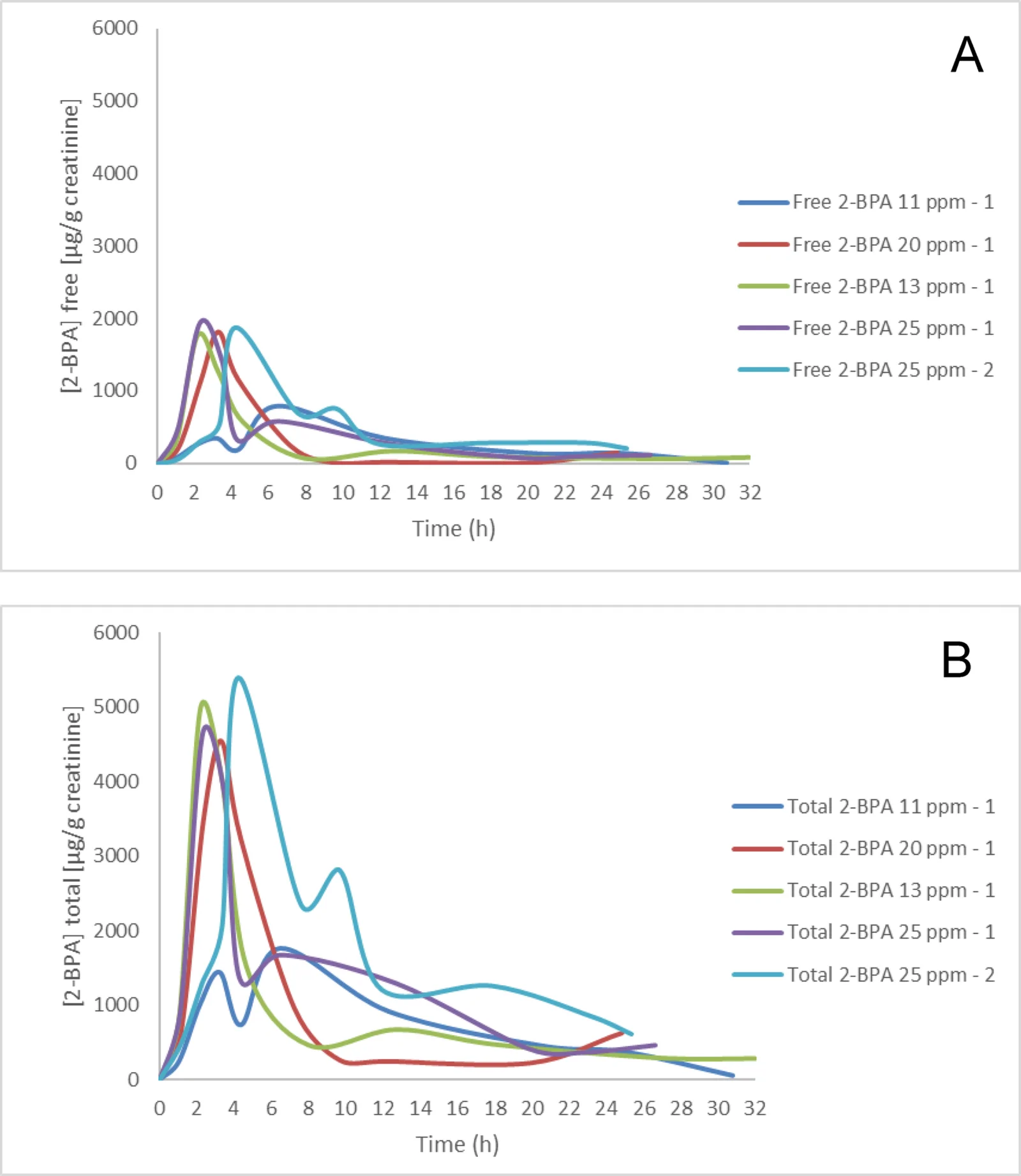
Urinary elimination kinetic curves of 2-BPA. Elimination curves are shown over time (x-axis) in h and the concentrations are expressed in µg/g creatinine (y-axis) for two researchers and four exposure concentrations. The pre-exposure was at 0 h. This was followed a 2-h exposure session. The two researchers were followed post-exposure for 32 h. For each graph, the legend states the air concentration corresponding to the curve and the participant identification number (1 or 2). **A** Free 2-BPA elimination kinetic curves in urine at different exposure concentrations. **B** Total 2-BPA elimination kinetic curves in urine at different exposure concentrations

To characterize urinary excretion patterns of 2-BPA, elimination kinetics were assessed following controlled PGBE exposure. Figure 5 presents creatinine adjusted urinary elimination kinetics for free (Fig. 5A) and total 2-BPA (Fig. 5B). Data are shown for two researchers in our laboratory exposed to 25 ppm PGBE for 2 h, and for one participant additionally exposed to 11, 13, and 20 ppm concentrations. These preliminary exposure experiments were used to evaluate if metabolites could be quantified in participant samples. Prior to exposure (0 h), free 2-BPA was undetectable in all urine samples. Free 2-BPA concentrations increased rapidly during the exposure period, peaking between the end of exposure and 2 h post PGBE exposure (Fig. 5A). Interestingly, a second peak was observed approximately 5 h after exposure ended. Trace levels of free 2-BPA were still detectable in urine samples collected 24 h post-exposure.

Total 2-BPA, quantified after an enzymatic deconjugation step and normalized to urinary creatinine concentrations showed similar kinetics (Fig. 5B). The amount of conjugated 2-BPA was approximately twice that of free 2-BPA. The concentration of total 2-BPA in urine increased at the start of the exposure and peaked between 2 and 4 h after exposure ended. A secondary peak was also observed between 4- and 8-h post-exposure. Notably, trace amounts of total 2-BPA remained detectable up to 30 h after exposure. These results indicate a biphasic elimination pattern for both free and total 2-BPA, with rapid absorption and delayed secondary peaks, suggesting possible enterohepatic recirculation or redistribution processes.

## Discussion

Our controlled human inhalation studies confirm that PGBE is metabolized to 2-BPA and PGME to 2-MPA, with metabolite formation detectable in urine in post-exposure. Our results are in line with previous PGME inhalation studies (Devanthéry et al. 2002; Hopf et al. 2012; Jones et al. 1997; Tomicic et al. 2011) and with biomonitoring data from occupational and environmental exposures (Ben-Brik et al. 2004; Fromme et al. 2013; Garlantézec et al. 2020; Laitinen 1997). Here, we provide the first in vivo evidence of 2-BPA formation from controlled PGBE exposure, supported by in vitro results using HepaRG cells.

We observed a mixture effect on urinary elimination when participants were co-exposed to PGME and PGBE, despite PGME being administered at 35 ppm, which is well below its OEL of 100 ppm. Notably, the air concentrations of each propylene glycol ether were identical in the single and combined exposure scenarios. However, the urinary concentrations of their respective metabolites, measured at the end of the 2-h exposure sessions, were consistently lower following the mixture exposure. Specifically, urinary 2-MPA concentrations were reduced by approximately 50% following exposure to the PGME/PGBE mixture (50 ng/mL), despite the airborne PGME concentration being only modestly lower compared to PGME-alone exposure (70 ng/mL). Similarly, urinary 2-BPA concentrations were approximately 20% lower following exposure to the PGME/PGBE mixture (230 ng/mL), despite only a modest reduction in airborne PGBE concentration compared to the PGBE-alone condition (280 ng/mL). Unlike PGME, for which the proportion of the PGMEβ impurity is regulated, no such regulatory limit exists for PGBEβ. The β-isomer content in PGBE can theoretically range from 35% to 50%, as its synthesis typically yields a mixture of mono-, di-, and tri-glycol ethers. These components, along with their respective isomers, are separated through successive distillation steps (de Ketttenis 2005). Currently, regulatory limits on β-isomer content apply to technical-grade PGME but not to other propylene glycol ethers.

Both solvents rapidly enter the bloodstream within three minutes of inhalation exposure in participants, and their absorption rates were not influenced by simultaneous inhalation of PGME and PGBE (Borgatta et al. 2022). Pulmonary diffusion, rather than active transport mechanisms, was identified as the primary route of uptake. PGME concentrations in blood continued to increase throughout the exposure period, consistent with its high blood: air partition coefficient and indicating extensive systemic absorption. In contrast, PGBE appeared to have a lower blood: air partition coefficient compared to PGME, suggesting a slower rate of absorption. It remains unclear whether pulmonary diffusion or enzymatic metabolism is the rate-limiting step in the conversion of PGBE to its metabolite, 2-BPA, as no in vitro time-course studies on 2-BPA formation currently exist. Notably, our study is the first to confirm that 2-BPA is indeed the primary metabolite of PGBEβ.

We demonstrated here that PGBE is metabolized to 2-BPA both in vitro and in vivo, thereby confirming earlier assumptions based solely on urinary biomonitoring data. In contrast, the metabolic conversion of PGME to 2-MPA has been previously established. (Werner et al. 2024) contributed mechanistic insight by quantifying the formation rate of 2-MPA in HepaRG 3D cultures and human liver S9 fractions; however, their study did not investigate PGBE or its metabolite, 2-BPA. Our in vitro experiments using HepaRG cells support the human exposure findings, showing that 2-BPA is specifically formed upon PGBE treatment, while PGME exposure yielded only trace 2-MPA levels. This is consistent with the known low β-isomer content in PGME. Importantly, CYP modulation experiments further suggested that CYP1A1 and/or CYP1A2 are involved in the metabolism of PGBE to 2-BPA, while other isoforms including CYP2E1, CYP2C19, CYP3A4, and CYP2A6 appear not to contribute significantly. The discrepancies between our findings and those of Werner et al. likely reflect differences in compound selection, exposure concentrations, or culture formats (e.g., 2D vs. 3D). Additionally, the potential for metabolic competition between PGME and PGBE, particularly at the level of ADH and ALDH enzymes, may explain the reduced metabolite formation observed during co-exposure. This mechanism has been previously described for structurally similar solvents (Tan et al. 2011). Although ADH and ALDH activity were not directly assessed in our study, Werner et al. (2024) highlighted their critical role in PGME metabolism, supporting this interpretation (Werner et al. 2024). Together, these findings highlight the complexity of solvent metabolism in HepaRG cells and emphasize the importance of specific CYP isoforms in determining the metabolic fate of glycol ethers. Future studies using complementary approaches, such as recombinant CYP expression systems, human liver microsomes, siRNA/CRISPR knockdown, or selective chemical inhibition, are needed to precisely identify the CYP enzymes responsible for PGBE and PGME metabolism.

Regulatory restrictions likely explain the difference in background exposure levels, as the β-isomer of technical grade PGME is limited to less than 5%, while no such restriction currently exists for PGBE (de Ketttenis 2005). Despite this regulation, 2-MPA levels were quantified in the majority of participants prior to the controlled exposure experiments. Importantly, participants were carefully selected to exclude individuals employed in occupations with known exposure to PGME or other related solvents. These findings suggest that exposure sources leading to 2-MPA formation are widespread in the general population, consistent with previous reports (Devanthéry et al. 2003; Garlantézec et al. 2013, 2020). This may reflect low-level, chronic exposure to consumer products or environmental sources containing trace amounts of PGMEβ or structurally related compounds. The persistence of such background levels underscores the importance of continued monitoring and re-evaluation of current regulatory thresholds, particularly for glycol ether isomers lacking specific restrictions.

In a child cohort study (*N* = 110), approximately 89% of the children had urinary 2-MPA concentrations above the LOD, indicating widespread background exposure (Garlantézec et al. 2020). Similarly, adults occupationally exposed to PGME exhibited urinary 2-MPA concentrations prior to the start of their work shifts that were comparable to those of non-exposed controls recruited from the same population(Ben-Brik et al. 2004). These findings suggest that the metabolite 2-MPA is not solely a marker of occupational exposure, but also reflects more diffuse, population-wide exposure. In our controlled experiments, 2-MPA was confirmed as a metabolite of PGMEβ. However, this metabolite is also formed following exposure to propylene glycol methyl ether acetate (PGMEA), an ester derivative of PGME that undergoes rapid hydrolysis in the body (ECETOC 2005). This shared metabolic pathway helps explain the frequent detection of 2-MPA in the general population, without a clearly defined source of exposure. While the general population may be exposed to low levels of PGME or PGMEA through consumer products, occupational settings often involve exposure to multiple industrial formulations, each with varying concentrations of these glycol ethers. These differences likely contribute to the variability observed in baseline urinary 2-MPA concentrations across populations. In contrast, PGBE and its derivatives are less commonly used in commercial and industrial contexts, which may explain why urinary 2-BPA levels were not reported in either of the aforementioned studies. As a result, the prevalence of 2-BPA in the general population remains largely unknown. A recent review provides a comprehensive summary of current knowledge regarding human exposure to propylene glycol ethers and their derivatives (De Luca and Hopf 2025), highlighting the need for further biomonitoring efforts to address these data gaps.

Biomonitoring studies assessing exposure to PGME and PGBE can quantify either the parent compound or their respective metabolites in urine. In our study, albeit with results from only two participants, we observed that urinary 2-BPA metabolite elimination peaked between the end of exposure and 2 h post-exposure, followed by a gradual decline. In contrast, the parent compound PGBE exhibited a much shorter apparent elimination half-life (Hopf et al. 2025 in preparation). This distinction between parent and metabolite kinetics can be advantageous for field biomonitoring, particularly when sampling is not immediate. A relevant example comes from a study conducted among graffiti removers working in Swedish underground stations and train depots. They were exposed to a mixture of organic solvents, including dipropylene glycol monomethyl ether (DPGME), propylene glycol monomethyl ether (PGME), and diethylene glycol monoethyl ether (DEGEE) (Anundi et al. 2000). Importantly, DPGME is metabolized to PGME, meaning both solvents share the same urinary metabolite, 2-MPA, which enables the assessment of combined exposure to PGME and DPGME through a single biomarker. Exposures were quantified in air with a maximum PGME value of 1.4 ppm (5.33 mg/m^3^) in depots and 8.4 ppm (32.78 mg/m^3^) in underground stations. While PGME itself was not detected in urine samples, the metabolite 2-MPA concentrations was present, with a mean urinary concentration of 0.72 mg/L (Anundi et al. 2000). Furthermore, 2-MPA concentrations were significantly higher in 38 graffiti removers compared to 18 unexposed office workers, but not significantly different from 10 unexposed depot workers. These findings highlight the value of 2-MPA as a sensitive biomarker for assessing PGME exposure, even at concentrations well below the OELs.

However, our controlled exposure study demonstrates that interpretation of biomonitoring data becomes complex when workers are exposed to multiple glycol ethers. At 35 ppm PGME, urinary 2-MPA reached 0.095 mg/L (~ 2.7 ng/mL per ppm), but co-exposure to 15 ppm PGBE reduced 2-MPA to 0.063 mg/L (~ 1.8 ng/mL per ppm). Similarly, 15 ppm PGBE alone yielded 0.305 mg/L 2-BPA (~ 20 ng/mL per ppm), while co-exposure reduced this to 0.251 mg/L (~ 16.8 ng/mL per ppm). These reductions of ~ 33% for 2-MPA and ~ 17% for 2-BPA indicate competitive metabolic inhibition and a non-linear dose-response. If urinary 2-MPA were used as a biomarker for PGME without recognizing PGBE co-exposure, which currently requires no labeling, PGME exposure would be underestimated, potentially leading to false conclusions that workers are below set exposure limits. This emphasizes the need for biomonitoring strategies that account for co-exposure effects when interpreting biomonitoring data. Although metabolic competition likely explains the reduced urinary metabolite elimination under co-exposure, a diffusion effect in the lungs has been suggested (Borgatta et al. 2022; He et al. 2025). This mechanism remains unconfirmed under mixture conditions and warrants further investigation.

OELs and occupational biological limit values (OBLs) are usually developed to protect workers handling hazardous substance (Hopf et al. 2024). OBLs can be developed from established OELs following harmonized TK modeling approaches (Hopf et al. 2025). While OELs are commonly set by regulatory authorities, the development of OBLs is less frequent, often hindered by insufficient human toxicokinetic data required to support their derivation. Expanding such evidence, particularly for widely used glycol ethers like PGME and emerging compounds like PGBE, is critical to strengthen occupational health risk assessments and regulatory guidance.

Further investigations are needed to better characterize occupational exposures to PGBE and to assess the effectiveness of using urinary 2-BPA concentrations as a biomarker for quantifying such exposures in workers routinely handling this solvent. Many occupational groups, such as cleaners, often have exposures to several propylene glycol ethers (Gerster et al. 2014), making it essential to investigate potential mixture effects and interactions. Notably, a recent in vitro study demonstrated that PGBE had the highest neurotoxic potential (Reale, 2023), underscoring its relevance for neurological risk assessment. Given these findings, research is needed to better evaluate the health risks associated with PGBE exposures, considering both occupational and general population.

## Conclusion

This study provides new insights into the toxicokinetics of PGBE and PGME exposure, demonstrating that 2-BPA is indeed formed in participants following controlled inhalation exposure to PGBE. Notably, simultaneous exposure to both PGME and PGBE resulted in reduced metabolism of their respective urinary metabolites, 2-BPA and 2-MPA. Additionally, in vitro experiments using HepaRG cells supported these finding by replicating human metabolite formation.

Given the widespread environmental and occupational exposure to glycol ethers, further research is needed to assess the neurotoxic potential of PGBE and the utility of urinary 2-BPA as a biomarker for exposure monitoring. Moreover, the observed interaction effects underscore the importance of assessing combined exposures to multiple glycol ethers, particularly in settings such as professional cleaning, to ensure adequate worker protection.

## Supplementary Information

Below is the link to the electronic supplementary material.

Supplementary Material 1

## Data Availability

All relevant data generated and analyzed during this study are presented in the main manuscript and supplementary material. The in vitro data, including cell viability, CYP induction, and metabolite formation in HepaRG cells, are provided in the supplementary material. Human exposure data from the controlled chamber experiments are fully reported in the manuscript. Due to ethical considerations and participant confidentiality, personal data from the exposure chamber study will not be shared. However, additional non-identifiable exposure-response data may be made available upon reasonable request to the corresponding author.
