# Supplementary material for: Urinary metabolite elimination from inhaled propylene glycol butyl ether (PGBE) and propylene glycol methyl ether (PGME) in healthy participants
Source: Arch Toxicol. 2026 Mar 10;100(6):2367–79. doi: 10.1007/s00204-026-04338-7 (PMC13658299; doi:10.1007/s00204-026-04338-7)
Supplement: Supplementary file 1 [file 204_2026_4338_MOESM1_ESM.docx]

**Supplementary file**

**Urinary metabolite elimination from inhaled propylene glycol butyl ether (PGBE) and propylene glycol methyl ether (PGME) in healthy human participants**

Nancy B. Hopf, Susanne Kaspar, Jamal Bouitbir, Jennifer Pache, Sylvain Le Gludic, Gregory Plateel, Nicole Charriere, Myriam Borgatta

**Materials and methods**

**Membrane toxicity**

Membrane toxicity was measured by the detection of adenylate kinase release, which results from the loss of cell membrane integrity. For adenylate kinase quantification, the ToxiLight assay kit (Lonza, Basel, Switzerland) was used accordingly to the manufacturer’s protocol. We used 0.4% Triton X as a positive control. Briefly, ToxiLight reaction buffer (100 μL) was mixed with 20 μL of supernatant from the treated cells in a new 96-well plate. After 5 min of incubation, the luminescence was measured with a Tecan M200 Pro Infinity plate reader (Männedorf, Switzerland). All data were normalized to positive control incubations containing 0.1% Triton X (set at 100 % cell lysis).

**Intracellular ATP content**

The intracellular ATP content, a marker for cell metabolic activity and viability, was determined using the CellTiter Glo kit (Promega, Madison, USA), following the manufacturers instruction. Briefly, 50 μL of assay buffer was added to each 96-well containing 50 μL culture medium. After incubation at room temperature for 15 min, luminescence was measured using a Tecan M200 Pro Infinity plate reader. All data were normalized to control incubations containing 0.1% DMSO.

**Levels of mRNA expression**

For RNA extraction and purification, the Qiagen RNeasy Mini Extraction Kit and DNA digestion kit (Qiagen, Hilden, Germany) were used according to the supplier’s protocol. RNA content and quality evaluation a NanoDrop 2000 (Thermo Scientific, Wohlen, Switzerland) was used. cDNA was synthesized from 1 μg RNA, using the Qiagen Omniscript system (Qiagen). Comparative real-time PCR quantifications were conducted using FastStart Universal SYBR Green Master from Roche (Rotkreuz, Switzerland) in a ViiA 7 Real Time PCR System from Applied Biosystems (Waltham, MA, USA). The relative quantities of the specifically amplified cDNAs were calculated by the comparative-threshold cycle method. As endogenous reference for mRNA determinations, glyceraldehyde 3-phosphate dehydrogenase (GAPDH) was used and no-template and no-reverse-transcription controls ensured the absence of non-specific amplification. The primers used were: *GAPDH* forward: 5’-AGC CAC ATC GCT CAG ACA C-3’, reverse: 5’-GCC CAA TAC GAC CAA ATC C-3’; *CYP1A1* forward: 5’-TCC AGA GAC AAC AGG TAA AAC A-3’, reverse: 5’-AGG AAG GGC AGA GGA ATG TGA T-3’; *CYP 2E1* forward: 5’-GAG GAT GTC GGC TAT GAC G-3’, reverse: 5’-ACT ATG GGA TGG GGA AAC AG-3’; and *CYP 2B6* forward:5’-ATG GG G C AC TGA AA A A GA CTG A-3’, reverse: 5’-AGA GG C G GG GAC AC T G AA TGA C-3’.

**Statistics**

Data are expressed as mean ± SD. Statistical analysis was performed using RStudio software (www.rstudio.com, RStudio 2013, version 2023-06.1+524) with the statistical language R (http://cran.r-project.org) (R Core Team, 2023). Statistical comparisons were performed using Kruskal-Wallis testing with Holm correction to account for multiple group comparisons. Statistical significance was set at * *p* ≤ 0.05.

**Figure S1. Intracellular ATP content and membrane toxicity in HepaRG cells after PGME and PGBE treatment with or without CYP induction.** (a) Intracellular ATP content in differentiated HepaRG cells following 24 h exposure to PGME or PGBE. (b) Adenylate kinase (AK) release as a marker of membrane toxicity under the same conditions. (c) Intracellular ATP content after three days of CYP induction with isoniazid, β-naphthoflavone, or phenobarbital, followed by PGME or PGBE treatment. (d) Membrane toxicity assessed as AK release under the conditions described in (c). Results represent mean ± standard error (SE) (n = 4). Ctrl, control; TX. Triton-X.

**Figure S2. CYP enzyme induction in HepaRG.** mRNA expression levels of a) CYP1A1, b) CYP2E1, and c) CYP2B6 were measured following three-day treatment with isoniazid, β-naphthoflavone, and phenobarbital, respectively. Results are presented as mean ± SE (standard error) (n = 4). Ctrl, control.

**Table S1. Overview of p-values adjusted.** Note: n.s. = all comparison within a group of increasing levels of either PGME was not significant

**Table S2. Summary of Kruskal-Wallis testing, multiple testing correction with Holm.** Note: n.s. = all comparisons withing a group of increasing levels of either PGME was not significant; *) for 2.5 mg/L, 25 mg/L, and 200 mg/L PGBE.

**Table S3. Summary of Kruskal-Wallis testing for 2-MPA, multiple testing correction with Holm.**

**Table S4. Summary of Kruskal-Wallis testing for 2-BPA, multiple testing correction with Holm.**
